# Comparison of Swing and Tilting Check Valves Flowing Compressible Fluids

**DOI:** 10.3390/mi11080758

**Published:** 2020-08-06

**Authors:** Zhi-xin Gao, Ping Liu, Yang Yue, Jun-ye Li, Hui Wu

**Affiliations:** 1 SUFA Technology Industry Co., Ltd., CNNC, Suzhou 215129, China; zhixingao@zju.edu.cn (Z.-x.G.); rgmbj20068@sina.cn (Y.Y.); lijy@chinasufa.com (J.-y.L.); wuh@chinasufa.com (H.W.); 2 Institute of Process Equipment, College of Energy Engineering, Zhejiang University, Hangzhou 310027, China

**Keywords:** check valve, Mach number, dynamic characteristics, computational fluid dynamics

## Abstract

Although check valves have attracted a lot of attention, work has rarely been completed done when there is a compressible working fluid. In this paper, the swing check valve and the tilting check valve flowing high-temperature compressible water vapor are compared. The maximum Mach number under small valve openings, the dynamic opening time, and the hydrodynamic moment acting on the valve disc are chosen to evaluate the difference between the two types of check valves. Results show that the maximum Mach number increases with the decrease in the valve opening and the increase in the mass flow rate, and the Mach number and the pressure difference in the tilting check valve are higher. In the swing check valve, the hydrodynamic moment is higher and the valve opening time is shorter. Furthermore, the valve disc is more stable for the swing check valve, and there is a periodical oscillation of the valve disc in the tilting check valve under a small mass flow rate.

## 1. Introduction

With the increasing demand for precise control, various kinds of valves are applied in the pipeline systems. Check valves, which are mainly used to prevent reverse flow, also have wide applications. The flow characteristics and the opening process of a check valve have great influences on the performance of the whole pipeline system [1,2].

During the check valve closure, the water hammer may appear damaging the pipeline. Yang et al. [3,4] focused on the water hammer problem caused by switching of parallel pumps, and they inhabited the phenomenon using a contra-motion check valve. Xu et al. [5] investigated a Contra-push check valve using computational fluid dynamics (CFD) methods; their results showed that valve slum did not occur, and the gap between plug and sleeve was a key factor determining the pressure-induced force. Ballun [6] used the reverse velocity and the deceleration to judge whether there would be a check valve slam in the water system, and seven kinds of check valves were experimentally tested under different deceleration. Lee et al. [7] found that valve chattering caused by acoustic resonance could lead to valve degradation. Lee and Leow [8] focused on the pressure surge when a check valve was closed under different flow conditions. Furthermore, some works focused on the dynamic characteristics of check valves. Marcinkiewicz et al. [9,10] conducted experiments using a swing disc and a tilting disc check valve, and the flow rate coefficient and the coefficient moment of hydrodynamic force were studied under different opening rates [9]. The effects of angular velocity and pressure were also investigated [10]; eventually a linear relationship between the pressure drop and the square of the inlet flow rate was found [9]. With the development of numerical computation, CFD becomes the most utilized method [11,12]. Leati et al. [13] focused on the dynamic characteristics of a ball check valve and a plate check valve in pipelines with high-frequency oscillation pumps using numerical and experimental methods, and the influence of valve design and fluid propagation effects were investigated. Knutson and Van De Ven [14] experimentally and numerically investigated the reed valves. Zhang et al. [15] focused on the flow force acting on the valve seat. Besides, Jin et al. [16] and Qian et al. [17,18] applied the Tesla valve, which is a new type of check valve, in hydrogen decompression, and the valve noise and the effects of valve stage on valve performance were investigated.

As the hydrodynamic moment acting on the valve disc of check valves is the key factor affecting the valve state and valve characteristics, works using experiments or CFD methods were carried out by many researchers. Leutwyler and Dalton [19] found that CFD methods could be used to predict the aerodynamic moment and lift in a butterfly valve over a wide range of pressure ratios, and the simulated results had good agreement with the tested results. Sibilla and Gallati [20] found that the CFD methods had a good ability to obtain accurate results of the hydraulic characteristics of a nozzle check valve. Li and Jim [21] studied the moment acting on the swing disk, and the moment acting on the fixed disk and the moment due to the disk rotation were obtained. Li et al. [22] used CFD methods to study the mechanism of cavitation in a bileaflet mechanical heart valve, which is a type of check valve. Lai et al. [23] focused on the opening period of a dual disc check valve using CFD and experimental methods, and they proposed a correlation to estimate the start and endpoints of disc rotations. Farrell et al. [24] proposed a method to calculate the required flow rate to achieve the fully open state or an intermediate opening of check valves, and their results can reduce the simulation work. Tran [25] presented a mathematical model to deal with the steady-state and dynamic closure process, which is useful for the selection of an economic check valve.

Recently, microvalves and microchannels have attracted a lot of attention [26,27,28,29]. Bui et al. [30] investigated an active check valve in a micropump. Ou et al. [31] proposed a microsphere-based check valve integrated into a micropump, which is designed for drug delivery applications. Qian et al. [32] focused on the nanoparticles flow in micro Tesla valves. Zhang and Zhang [33] also investigated a passive check valve.

To date, nearly all work on the dynamic characteristics of check valves focuses on incompressible fluids like water, while compressible fluids have been paid little attention. However, in some cases, high-temperature water vapor may be the working fluid, thus, it is of importance to study check valves flowing compressible fluids. In this paper, a numerical comparison between a swing check valve and a tilting check valve is carried out. The influences of the valve opening and the mass flow rate are investigated. The maximum Mach number, the valve opening, and the hydrodynamic moment acting on the valve disc are evaluated to compare the steady and dynamic characteristics.

## 2. Numerical Methods

### 2.1. Physic Model

**Figure 1:**
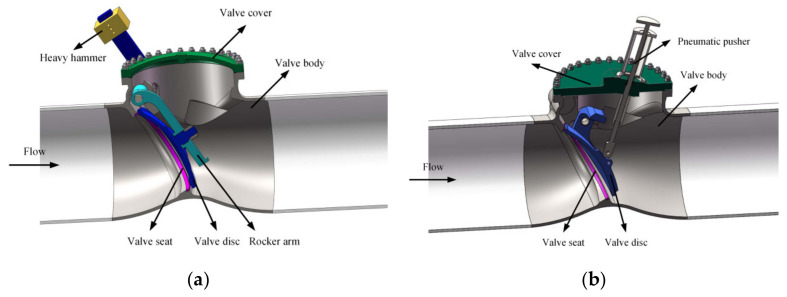
The structure of the investigated check valves: (**a**) the swing check valve; (**b**) the tilting check valve.

The structure of the investigated check valves is shown in Figure 1, and the check valves mainly consist of the valve body, the valve seat, the valve disc, the valve cover, and the heavy hammer. The valve upstream pipe length is 4D and the downstream pipe length is 10D.

### 2.2. Numerical Model

Different mass flow rates with constant outlet pressure are focused to investigate the dynamic characteristics of the two types of check valves. The working fluid is water vapor under 159.3 °C and is considered as ideal gas due to its high Mach number at a small valve opening. The Reynolds number of check valves is around 1 × 10^6^, which means that there is a high-intensity turbulent flow. Thus, the realizable *k*-*ε* turbulence model is adopted to solve the flow fields together with the Navier–Stokes equations and energy equation. The corresponding equations are as follows:

(1) $$\frac{\partial }{\partial x_{j}}\rho u_{j}=0$$

(2) $$\frac{\partial }{\partial x_{j}}\left(\rho u_{i}u_{j}+p\delta_{ij}-\tau_{ij}\right)=0$$

(3) $$\frac{\partial }{\partial x_{j}}\left(\rho u_{j}C_{v}T+u_{j}p+C_{p}\frac{\mu }{\mathrm{Pr}}\frac{\partial T}{\partial x_{j}}-u_{i}\tau_{ij}\right)=0$$

where *ρ* is the water vapor density, *u* is the water vapor velocity, *p* stands for pressure, *μ* stands for the dynamic viscosity of water vapor, *C*_v_ and *C*_p_ stand for specific heat, Pr stands for the Prandtl number, and *τ*_ij_ stands for viscous stress. As for the realizable *k-ε* turbulence model, it has been described in our previous study [34,35,36].

To investigate the dynamic characteristics of check valves, the common methods used by other researchers are the fluid–solid interaction (FSI) method, the dynamic mesh method [21,22,23], and the weighted-essentially non-oscillatory (WENO) method [37,38]. The dynamic mesh method is adopted in this study to solve the opening period of check valves. During the movement of the valve disc, the relationship between the resultant moment and the valve opening angle is shown below:

(4) $$M=I\frac{d^{2}\left(\alpha +\theta \right)}{dt^{2}}$$

where *M* = *M*_g_ + *M*_h_ + *M*_f_ is the resultant moment acting on the valve disc, *M*_g_ stands for the gravity moment, *M*_h_ stands for the hydrodynamic moment, and *M*_f_ stands for the friction moment. *I* stands for the moment of the inertia of the valve disc. *α* stands for the angle of the valve seat. *θ* stands for the valve opening angle. For the investigated swing check valve, the maximum valve opening is 45°, while for the investigated tilting check valve, the maximum valve opening is 50°.

All governing equations are solved by the commercial software Fluent which is based on the finite volume method. The density-based implicit algorithm is utilized, and the second-order upwind spatial discretization method is adopted to solve the flow, the turbulent kinetic energy, and the turbulent dissipation rate equations. Mass flow outlet, pressure inlet, and no-slip wall are set as the boundaries. Although there are no direct experiments about the water vapor flow inside check valves, the methods applied in this study have been proved in other work. Chen et al. [39] and Qian et al. [40] studied the compressible superheated steam flow in pressure-reducing valves.

**Figure 2:**
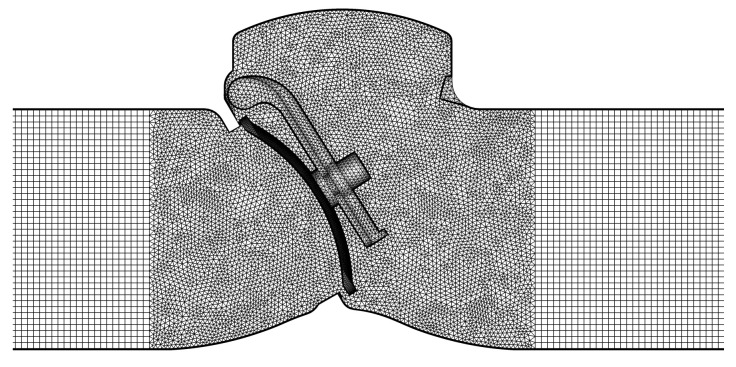
Grid of the swing check valve.

Because the structure of the two types of check valves is symmetrical, a half model is used to decrease the simulation time. The hybrid grid with the boundary layer is generated and is shown in Figure 2. The grid is separated into three zones, namely, the boundary layer zone close to the wall of the valve body, the boundary layer zone close to the wall of the valve disc, and the rest. During the simulation, the boundary layer zone close to the wall of the valve disc is assumed to move with the valve disc.

**Figure 3:**
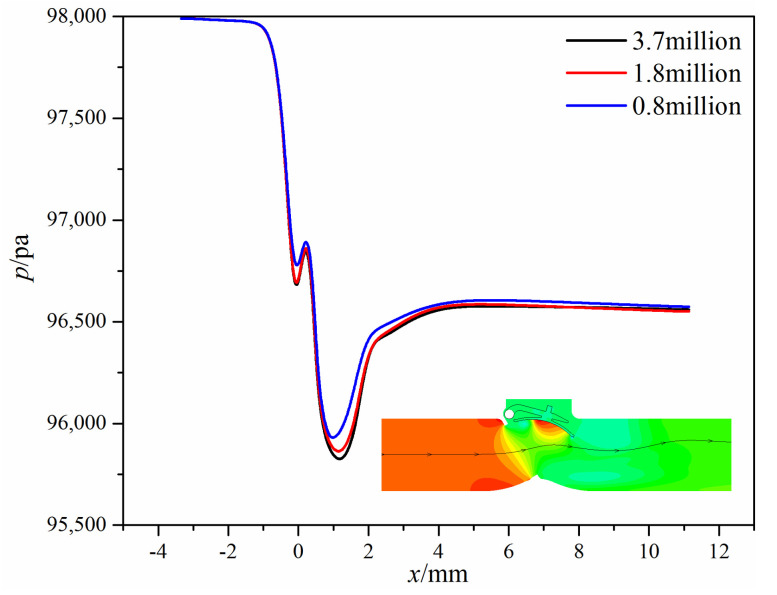
Pressure distribution along the same streamline under different grid numbers.

To eliminate the effects of the grid number, the results of three different grids are compared. Figure 3 shows the pressure distribution along the same streamline when the swing check valve reaches the fully open state. Table 1 shows the pressure difference between the inlet and the outlet under different grid numbers. Together with Figure 3 and Table 1, it can be inferred that when the grid number is above 1.8 million, the results barely change; thus, the second grid generated method is applied eventually.

## 3. Results

Under small valve openings, there is a limited flow area between the valve seat and the valve disc, which is on the order of millimeters. In this section, the pressure, temperature, and velocity distributions under different mass flow rates at a steady-state are obtained and compared firstly, and then the opening process is focused to compare the valve dynamic characteristics.

### 3.1. The Swing Check Valve

**Figure 4:**
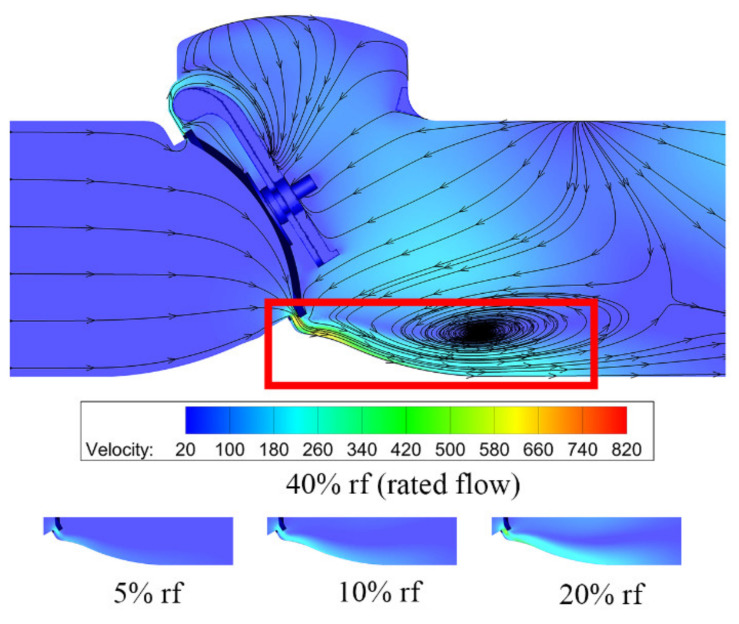
Velocity distributions under different mass flow rates.

When the valve opening is 4%, the velocity distributions on the symmetry plane of the swing check valve is shown in Figure 4. After water vapor flows through the valve seat, there is a high-speed jet, as marked, and the maximum velocity nearly reaches 12 times the inlet velocity, resulting in a high Mach number. An obvious vortex can also be found at the bottom of the swing check valve, which is because of the high-speed jet. At the top of the swing check valve, the cross-section area is smaller than the bottom of the swing check valve, and water vapor flows along the surface of the valve disc because of the valve structure. Velocity distributions under different mass flow rates are also shown in Figure 4. Here, only the velocity distributions with large variations are compared. It can be found that the influence of the high-speed jet increases with the mass flow rate.

**Figure 5:**
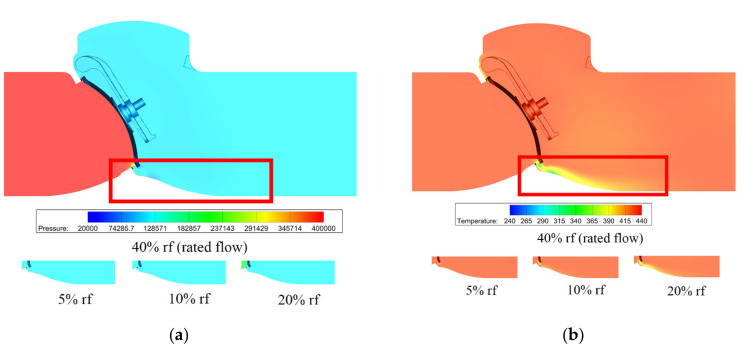
Pressure and temperature distributions under different mass flow rates: (**a**) pressure distribution; (**b**) temperature distribution.

Figure 5 shows the pressure and temperature distributions under different mass flow rates. When the mass flow rate is smaller than the 10% rated flow, the pressure variation is very small, thus there is a small variation in temperature. As the mass flow rate increases, obvious pressure difference appears and the maximum pressure difference reaches 0.4 MPa when there is a 40% rated flow. From the temperature distributions, one can find obvious temperature variation under a high mass flow rate, and the maximum temperature difference reaches 200 K.

**Figure 6:**
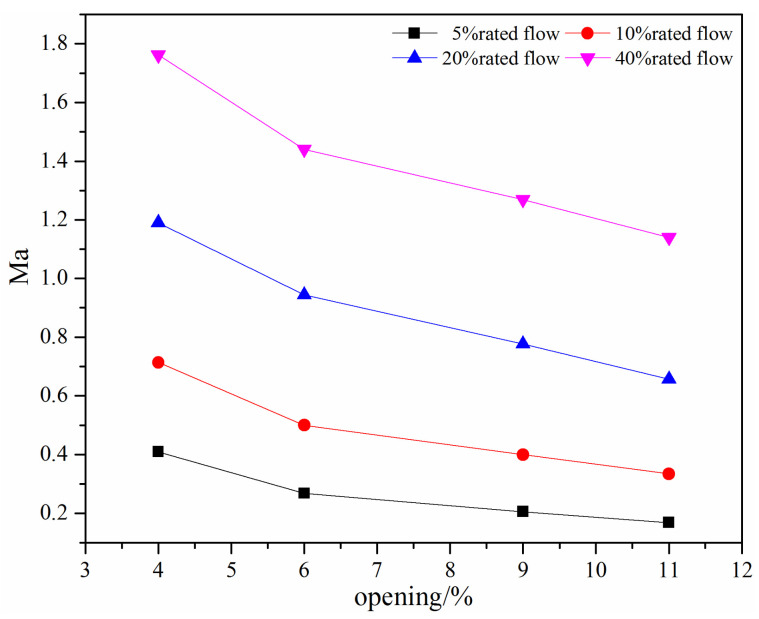
The maximum Mach number under different mass flow rates at small valve openings.

For the compressible flow, the Mach number is an important dimensionless number and is related to the aerodynamic noise and flow stability. As for the swing check valve and the tilting check valve, when there is a small valve opening, the Mach number is usually high. Figure 6 shows the maximum Mach number under different mass flow rates at small valve openings for the swing check valve. It can be seen that the Mach number increases with the decrease in the valve opening and the increase in the mass flow rate.

From Figure 6, the Mach number is large than 0.3 except when the mass flow rate is 5% rated flow, which means the water vapor should be considered as an incompressible fluid. When the mass flow rate is above 20% rated flow, the Mach number is close to 1 and eventually larger than 1, which means there may exist large noise and instability flow.

For check valves, their valve opening dynamics are important in the applied piping system. Here, the valve opening time and hydrodynamic moment acting on the valve disc are investigated to obtain the key valve characteristics of the swing check valve.

**Figure 7:**
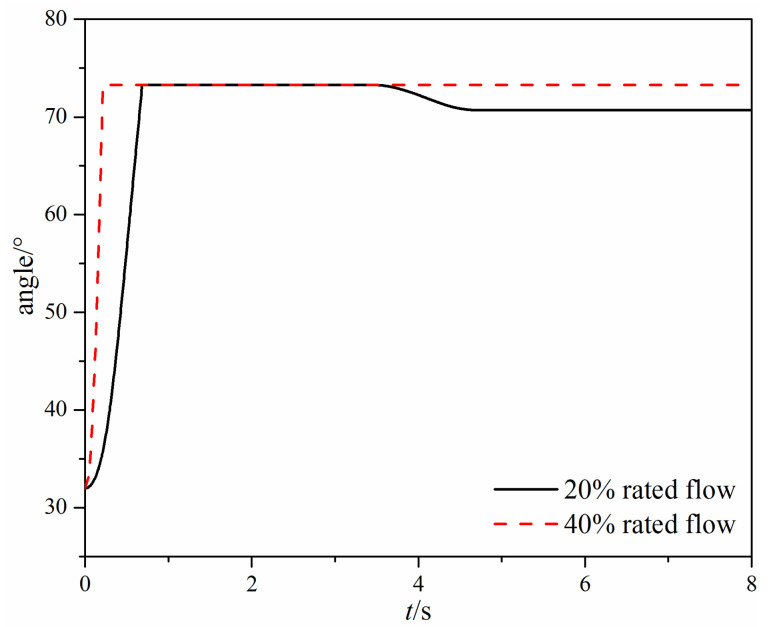
The time-varying opening angle under different mass flow rates.

Figure 7 shows the time-varying opening angle of the swing check valve under different mass flow rates. It can be found that when there is a small mass flow rate, the swing check valve reaches the fully open state around 0.7 s firstly, then the valve opening begins to decrease gradually at 3.4 s, and finally the valve disc stabilizes at a 94.3% valve opening. When there is a large mass flow rate, the swing check valve reaches the fully open state rapidly and then remains in the fully open state.

**Figure 8:**
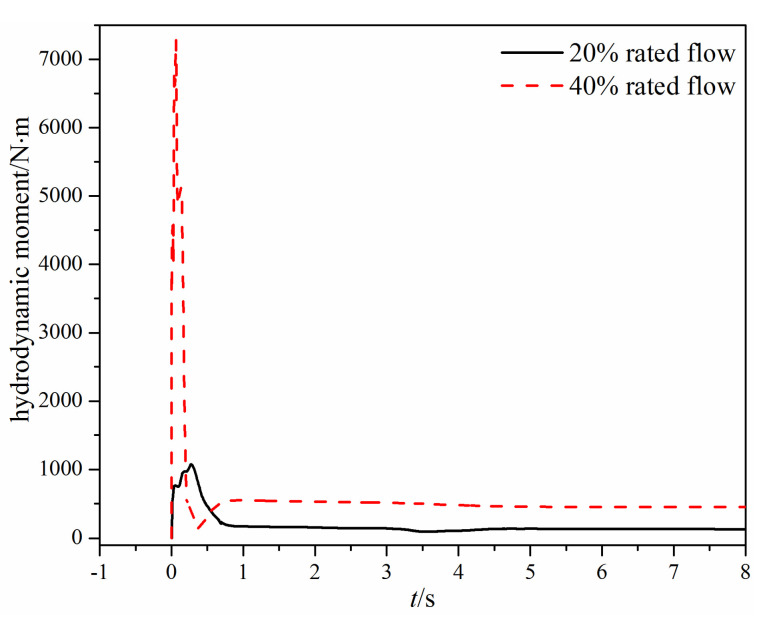
The time-varying hydrodynamic moment under different mass flow rates.

Figure 8 shows the time-varying hydrodynamic moment acting on the valve disc under different mass flow rates. When there is a large mass flow rate, the hydrodynamic moment first increases rapidly and reaches the maximum when the valve opening is about 44%. Then, the hydrodynamic moment decreases rapidly, and a small variation appears before the hydrodynamic moment reaches a stable value. The large hydrodynamic moment leads to the swing check valve reaching a 100% valve opening at 0.22 s, which can be seen in Figure 8. When the mass flow rate is small, the change rate of the hydrodynamic moment is small compared with the large mass flow rate, and the ratio of the maximum hydrodynamic moment under two different mass flow rates is more than 7.

### 3.2. The Tilting Check Valve

**Figure 9:**
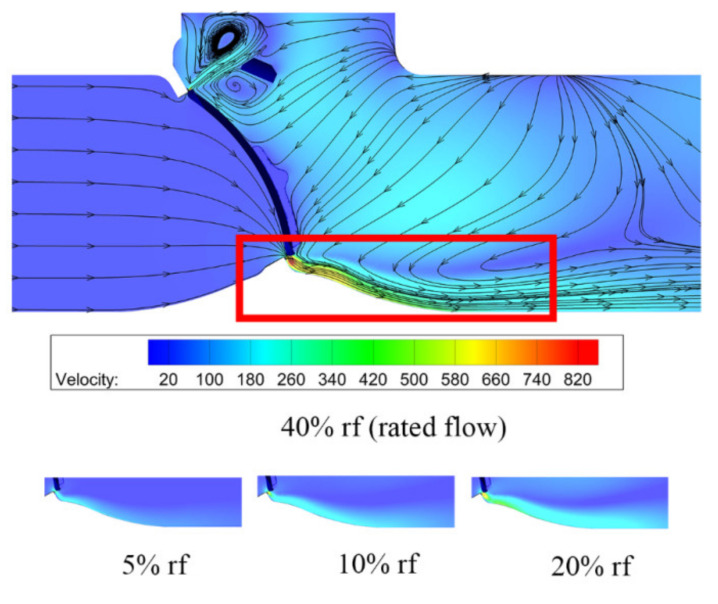
Velocity distributions under different mass flow rates.

When the valve opening of the tilting check valve is 4%, velocity and streamline distributions on the symmetry plane are as shown in Figure 9. Compared with the swing check valve, the maximum velocity is higher and there is no vertex at the bottom of the tilting check valve due to the structure difference of the valve seat and the valve disc.

**Figure 10:**
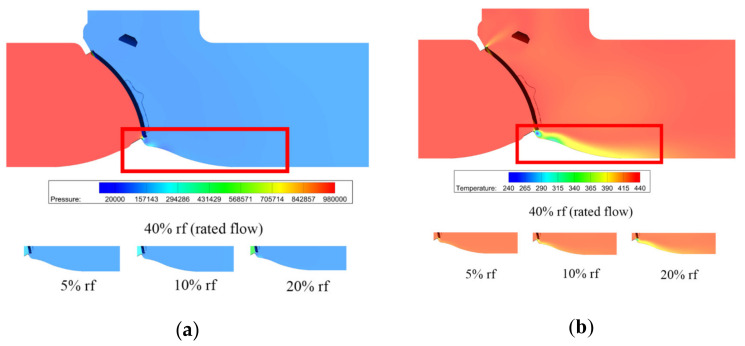
Pressure and temperature distributions under different mass flow rates: (**a**) pressure distribution; (**b**) temperature distribution.

The pressure and temperature distributions under different mass flow rates are shown in Figure 10. Compared with Figure 5, the pressure difference is higher in the tilting check valve, and the maximum pressure difference is nearly two times that of the swing check valve, while for the temperature, no obvious difference can be found.

**Figure 11:**
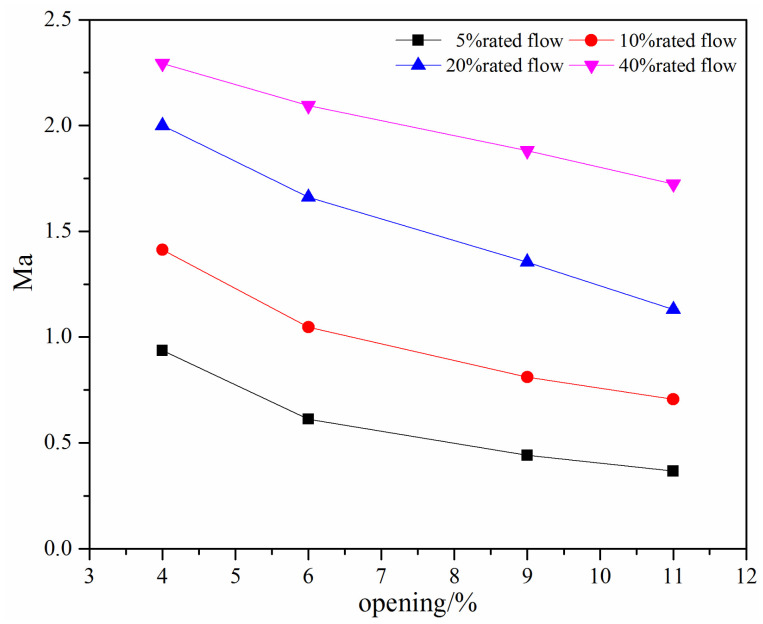
The maximum Mach number under different mass flow rates at small valve openings.

The maximum Mach number under different mass flow rates at small valve openings is shown in Figure 11. The maximum Mach number decreases with the increase in the valve opening and decrease in the mass flow rate. For the investigated mass flow rate, the maximum Mach number is generally above 0.3, indicating a compressible flow in the tilting check valve. When the mass flow rate is above the 20% rated flow, the maximum Mach number is above 1, which means there is a supersonic flow or even severe noise.

**Figure 12:**
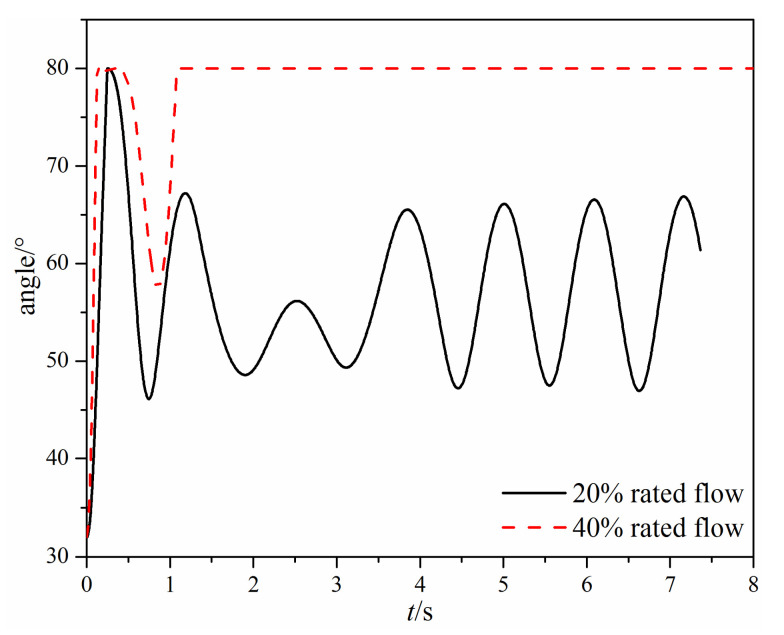
The time-varying opening angle under different mass flow rates.

The time-varying valve opening angle of the tilting check valve is shown in Figure 12. When there is a small mass flow rate, the valve disc first reaches 100% opening at 0.25 s; then, the valve disc rotates toward the valve seat and starts to oscillate. Finally, the valve disc keeps oscillating at a constant frequency. While there is a large mass flow rate, the valve disc reaches 100% opening at 0.125 s, and then the valve disc starts to rotate backward at 0.345 s. However, different from the small mass flow rate, the valve disc finally keeps at the 100% opening after 1.08 s.

**Figure 13:**
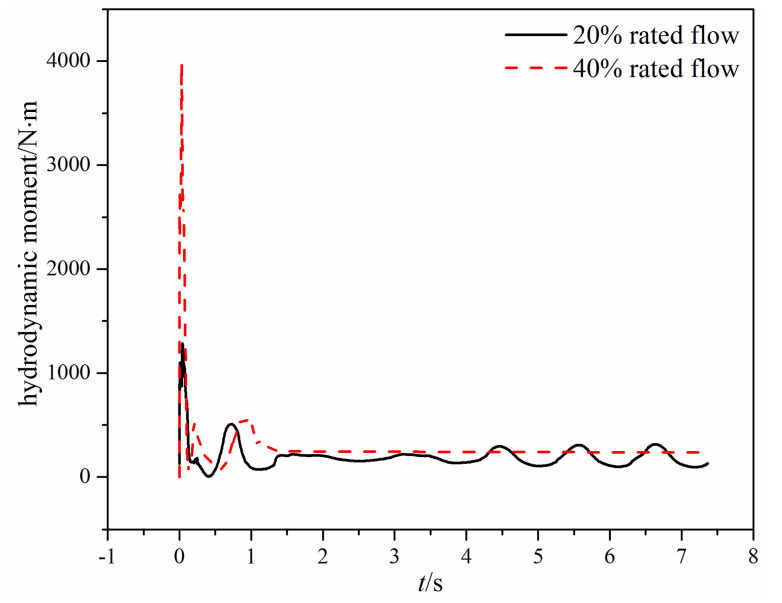
The time-varying hydrodynamic moment under different mass flow rates.

Figure 13 shows the time-varying hydrodynamic moment acting on the valve disc of the tilting check valve under different mass flow rates. When the mass flow rate is small, the hydrodynamic moment fluctuates eventually because of the inertia of the valve disc. When the mass flow rate is large, the hydrodynamic moment will stabilize eventually because the valve disc reaches the fully open state. From Figure 13, it can also be found that when the mass flow rate is 40% rated flow, the maximum hydrodynamic moment during the valve opening is nearly four times that under the 20% rated flow.

## 4. Discussion

The difference between the swing check valve and the tilting check valve is obvious. From the velocity and pressure distributions Figure 4, Figure 5, Figure 9 and Figure 10 one can find that the velocity and pressure are higher in the tilting check valve under the same conditions, and there also exist differences in terms of streamline between the two different types of check valves.

The Mach number, which is used to stand for the compressibility or the potential of the occurrence of the aerodynamic noise, is compared at small valve openings. From Figure 6 and Figure 11, it can be found that the variation trend of the maximum Mach number with the valve opening and the mass flow rate is the same; that is, the maximum Mach number increases with the decrease in the valve opening and the increase in the mass flow rate. However, the maximum Mach number in the tilting check valve is higher. For the tilting check valve, when the mass flow rate is of 10% rated flow, the maximum Mach number is even slightly higher than that in the swing check valve with a 20% rated flow. The reason is that the rotary center of the swing check valve is higher than the tilting check valve, which makes a relatively large cross-section.

When the mass flow rate is large, the comparison of the time-varying opening angle and the hydrodynamic moment of the two different types of check valves can be seen in Figure 7, Figure 8, Figure 12 and Figure 13. The main difference is that the swing check valve is more stable, and the hydrodynamic moment in the swing check valve is also higher. At the fully open state, the hydrodynamic moment in the swing check valve is about 453 N·m, while the hydrodynamic moment in the tilting check valve is 244 N∙m.

When the mass flow rate is small, the valve disc in the tilting check valve rotates quicker than the swing check valve, but the hydrodynamic moment cannot resist the gravity moment, so the valve disc cannot maintain the fully open state and thus begins fluctuating. Eventually, the valve opening of the tilting check valve varies from 58.8% to 83.8%. For the swing check valve, after the valve disc reaches the fully open state, the valve disc remains still for about 3 s, and then the valve disc begins to rotate backward.

The hydrodynamic moment in the tilting check valve is higher at first, which leads to quicker movement than the swing check valve. However, the hydrodynamic moment decreases rapidly subsequent, and when it is smaller than the gravity moment, the valve disc rotates backward, subsequently increasing the hydrodynamic moment. Thus, there is a fluctuating hydrodynamic moment. In contrast, for the swing check valve, although the hydrodynamic moment also decreases after it reaches the maximum value, the valve disc maintains the fully open state because of the large friction moment (183 N∙m), which is helpful for the stability of the compressible water vapor flow.

## 5. Conclusions

In order to gain a better understanding of the swing check valve and the tilting check valve with the same valve body, the computational fluid dynamics method was utilized to investigate their millimeter-scale flow characteristics and dynamic opening characteristics. Under small valve openings, velocity and pressure in the tilting check valve are higher. The maximum Mach number in the two types of check valves increase with the decrease in the valve opening and the increase in the mass flow rate. The maximum Mach number in the tilting check valve is higher than that in the swing check valve, and the maximum Mach number of the 10% rated flow in the tilting check valve is higher than that of the 20% rated flow in the swing check valve. During the opening process, the swing check valve is more stable, and the hydrodynamic moment acting on the valve disc of the swing check valve is higher. At a small mass flow rate, the valve disc of the tilting check valve fluctuates between 58.8% and 83.8%, while the opening of the swing check valve is 94.3%. At a larger mass flow rate, there is an oscillation of the valve disc of the tilting check valve before it finally reaches the fully open state, while there is no oscillation in the swing check valve. This work may be useful for the design of swing and tilting check valves with other dimensions.

## Figures and Tables

**Table 1:** The pressure difference between the inlet and the outlet under different grid numbers.

Grid Number × 10^6^	Pressure Difference/Pa	Error/%
0.8	1419.20	−1.6%
1.8	1442.69	-
3.7	1433.62	−0.63%
